# Investigating the Efficacy of HPV Vaccines in Preventing Cervical Cancer from 2006 to 2018 in the US: A SEER Data Set Analysis

**DOI:** 10.2174/1574887118666230410093715

**Published:** 2023-08-15

**Authors:** Alexander Mattis, Hind Beydoun, Yuliya Dobrydneva, Rohini Ganjoo

**Affiliations:** 1 Department of Biomedical Laboratory Science, School of Medicine and Health Sciences, The George Washington University, Ashburn VA, USA;; 2 Department of Research Programs, Fort Belvoir Community Hospital, Fort Belvoir, VA, USA;; 3 School of System’s Biology, George Mason University, Fairfax, VA, USA

**Keywords:** Human papillomavirus, cervical cancer, vaccine efficacy, cancer incidence, SEER, alternative endpoints, sexually transmitted infection

## Abstract

**Background:**

Human papillomavirus (HPV) is the most common sexually transmitted infection in the US.The first HPV vaccine was introduced in 2006. There are three different HPV vaccines that commonly target high-risk HPV types.

**Objective:**

This study compares HPV vaccine efficacy based on alternative endpoints with the most recently available cervical cancer incidence data from the Surveillance, Epidemiology and End Results (SEER) program and SEER*Stat statistical software.

**Methods:**

The incidence of cervical cancer, mined from the most recent April 2021 SEER data set, was stratified according to age and racial groups. Trend analysis reporting cervical cancer incidence percentage change (PC) and annual percentage change (APC) was calculated by SEER*Stat statistical software.

**Results:**

A total of 46,583 cases of cervical cancer were reported, with an average of about 3,580 incidents of cervical cancer per year, with an overall decrement of about 60 cases over the period of 12 years. The percentage change according to age and race groups varied between -15.9 among 40-44 years old (yo) and +13.8 among 30-34 yo, and from -12 among non-Hispanic White women to +13 among Hispanic women. Statistically significant APC was observed for five of the nine age groups and four of the five racial groups.

**Conclusion:**

There seems to be little if any, correlation between cervical cancer incidence and the HPV vaccine program in the US. HPV vaccine efficacy based on alternative endpoints, such as nucleic acid testing and cytological, surgical, and seropositivity endpoints, is fair. Therefore, it is important to emphasize such alternative testing and surrogate endpoints.

## 1 INTRODUCTION

The human papillomavirus (HPV) is a small double-stranded circular DNA virus that causes the most common sexually transmitted infection in the US. Persistent HPV infections of the mucosa epithelium are the causative agent of various anogenital and oropharyngeal cancers. Approximately 80% of all HPV diseases are associated with cervical cancer manifestations, and over 99% of cervical cancers are caused by HPV [1]. There are about 200 types of HPV, of which more than 40 invade the urogenital tract. All infective HPV types are subdivided into high and low risk groups based on their oncogenic properties. Only the high-risk types of HPV (HRHPV) develop into cancer. HRHPV types 16 and 18 are the most virulent and cause about 70% of all invasive cervical cancer worldwide. There are 16 other HRHPVs that account for the remaining 30%. Low-risk HPV (LRHPV) types 6 and 11, although not oncogenic, are associated with the development of condylomas (genital warts) [2, 3].

HRHPV infections follow a predictably slow and reversible development pattern into a cancerous tumor. The four steps in cervical cancer development are infection ofthe epithelial transformation zone of the cervix, viral persistence, progression of a clone of persistently infected cells to a precancerous lesion, and finally, invasion of that lesion through the basement membrane of the cervical epithelium. These four steps can take years to over a decade to occur and the host immune system clears most infections and precancerous lesions. Specifically, 90% of HRHPV infections are cleared over 84 months [4].

### 1.1 HPV Vaccines

First introduced in the United States (US) in 2006 for the primary prevention of cervical cancer, three different HPV vaccines are currently FDA licensed, commonly targeting HRHPV types 16 and 18. All three vaccines are composed of manufactured virus-like particles representing the L1 protein subunit of HPV. The first bivalent vaccine, Cervarix, targets only HRHPV type 16/18. The second and most widely used is the quadrivalent vaccine Gardasil, which targets HRHPV type 16/18 in addition to LRHPV type 6/11. Finally, the most recent nine-valent vaccine (that has replaced the four-valent type), Gardasil9, additionally targets HRHPV types 31/33/45/52/58 [3, 5]. All three HPV vaccines are intended to be administered prophylactically at a pre-sexual age. The CDC recommends two vaccine doses for males and females 9-15 years old (yo) and three doses up until 26 [6].

### 1.2 Determining HPV Vaccine Efficacy

The gold standard for judging vaccine efficacy is its prevention of the disease end-state. In this case, cervical cancer frequency in young girls and women provides the best picture of overall HPV vaccine efficacy. And therein lies the problem. According to the most recent cervical cancer screening recommendations, the effect of the HPV vaccine on cervical cancer is not yet characterized [7]. Cervical cancer can take over a decade to develop and vaccines were first approved for use in the US in 2006, thus the data needs to be continually updated to yield the most accurate representation of HPV vaccine efficacy.

This research aims to characterize national trends in cervical cancer frequency to assess the impact of HPV vaccines. This study reports HPV vaccine efficacy based on alternative endpoints and determined trends in US cervical cancer frequency between 2006 and 2018 using Surveillance, Epidemiology, and End Results program (SEER) data.

## 2 MATERIALS AND METHODS

### 2.1 Data Source

Cancer incidence data were exclusively used from the Surveillance, Epidemiology, and End Results (SEER) program maintained by the National Cancer Institute. SEER is a leading source of cancer incidence and survival data in the US and is collected from 21 national population-based registries that cover approximately 34.6% of the population. The program collects data on patient demographics, primary tumor site, tumor morphology and stage at diagnosis, first course of treatment, and follow-up for vital status. Geographic incidence data has been included in some of the recent data sets. However, it is localized to 16 different regions and was incomplete for the purposes of this research. SEER data is available in two different products; SEER Research and SEER Research Plus. The latter, which was utilized for this research, was recently created to protect against the risk of patient re-identifiability and requires authentication through an institutional account [8]. For the purposes of access, George Washington University sponsored this project and provided principal investigator credentials through an Electronic Research Administration (eRA) commons account. This research activity was determined to be exempt from 32 CFR 219 since the SEER data is publicly available and does not contain any identifiable patient information, and is thus exempt.

### 2.2 Patient Selection

All incidence data was mined from the current SEER data set, submitted November 2020, released April 2021, with data representing 21 cancer registries. The specific data set used for this research is titled “Incidence - SEER Research Plus Limited-Field Data, 21 Registries, Nov 2020 Sub (2000-2018)”. Case selection of the data was selected for the site and morphology of the cervix uterus, based on ID-0-3/WHO 2008, and specifically included female gender, all defined races/ethnicities (non-Hispanic white, non-Hispanic Black, non-Hispanic American Indian/Alaska Native, Non-Hispanic Asian or Pacific Islander, Hispanic), while unknown race/ethnicity was excluded. A 2006-2018 incidence date range was selected because HPV vaccines were first introduced in 2006 and the last available data is for 2018. The user-defined age range, available in 5-year increments, was set to 25 through 64 years of age based on CDC vaccination guidelines, 2006 vaccine rollout, and the earliest cervical cancer incidence. The CDC recommends HPV vaccination for people 9 to 26 yo and clinical decisions about vaccination for 27 to 45 yo individuals [6]. Age range 60 to 64 was added as a comparative control.

### 2.3 Statistical Methods

All statistical analyses were performed using the SEER data set compatible statistical software SEER*Stat version 8.3.9. Incident cases of cervical cancer occurring between 2006 and 2018 were stratified according to two demographic characteristics (age and race/ethnicity), by cross-tabulating the year of diagnosis against age group and the year of diagnosis against race/ethnicity. Trends in cervical cancer incidence were estimated using percent change (PC) and annual percent change (APC), while applying the same data search specifications mentioned above. SEER*Stat calculates PC rates over a specified period by taking the difference between the initial and end rate. This difference, divided by the initial rate multiplied by 100, results in the PC. In this study, a one-year period was assigned to the PC algorithm. SEER*Stat also calculates APC by using calendar year as a regressor variable, while fitting a least squares regression line to the natural logarithm of rates. The standard error for APC is calculated based on the fit of the regression line. The significance of APC was determined using “APC to 0” and “APC to APC” significance tests [9]. All frequency tables and PC/APC calculations generated by SEER*Stat were transferred to Excel for formatting and graphical manipulation. Two-tailed statistical tests were evaluated at α = 0.05.

## 3 RESULTS

There was a total of 46,583 reported cases of cervical cancer for patients aged 25-64 yo between 2006 and 2018 (Table **1A**). The highest incidence was reported in 2008 (3,684) and the lowest was in 2013 (3,400). As expected, there is a peak of age-related incidence between the ages of 40-44 (7,393) and 45-49 (7,240) yo. The lowest incidence was between the ages of 25 -29 (2,461) can be attributed to the transmission of sexually transmitted disease and HPV’s slow pattern of oncogenesis (Table **1A**, Fig. (**1**). That same data set reported a total of 46,157 cervical cancer cases delineated by race (Table **1B**). There were 24,492 non-Hispanic white cases, followed by 10,695 Hispanic (all races), and 6,522 non-Hispanic black. Non-Hispanic American Indian/Alaska Native had the least number of cases (377) (Table **1B**).

The lined scatter plot in Fig. (**2**), represents total cervical cancer incidence per year, revealing a substantial dip in reported cases between 2008 and 2015. While the highest incidence of cervical cancer based on year of diagnosis and age was reported to be 3,684 in 2008, the lowest incidence was 3,400 in 2013. The slope of the trend line applied to that scatter plot is -1.8736 (from y = -1.8736x + 7353) with a weak squared correlation coefficient of R^2^ = 0.0061. Next, we analyzed the annual cervical cancer incidence based on race. More than 53% of all cases reported, annually were from non-Hispanic white females, following which the rates of cervical cancer incidence decreased, respectively, from Hispanic, to non-Hispanic Black, to non-Hispanic Asian or Pacific Islander, and Non-Hispanic American Indian or Alaska Native Table **1B**, Fig. (**3**).

As seen in Table **2A**, the PC for total cervical cancer incidence from age 25-64 yo between 2006 and 2018 is -1.7. For the incremental age groups, the PC varies from -15.9 at 40-44 yo to +13.8 at 30-34 yo. The racial distribution of PC varies from -12 for the non-Hispanic white group to +13 for the Hispanic group. Further, from Table **2B**, we observe that five of nine age groups had statistically significant APC: 30-34 yo was +1.5, 40-44 yo was -1.4, 45-49 yo was -1.3, 55-59 was +1.3, and 60-64 yo was +1.4. Four out of five racial groups had statistically significant APC: non-Hispanic white was -0.7, non-Hispanic Black was -0.7, non-Hispanic Asian or Pacific Islander was +1.8, and Hispanic was +0.9.

## 4 DISCUSSION

This paper presents findings from SEER data for cervical cancer incidence for women aged between 25 - 64 years over a period of 12 years (2006 - 2018). Results indicate that the highest number of cases were observed in 2008 and among the races, non-Hispanic white women had the highest incidence. Based on the incidence data in this study, there seems to be little, if any, correlation between cervical cancer incidence in women and young girls and the HPV vaccine program in the US.

Based on the analysis of alternative endpoints, HPV vaccines are efficacious and there should be a measurable impact on cervical cancer incidence over a decade after the vaccines were introduced. Logically, the decreased cervical cancer incidence distribution across age groups should follow the official CDC vaccination schedule based on a 2006 start date. Vaccinations are recommended for children as early as 9 yo through age 26 yo. Clinically-based decisions are recommended from ages 27- 45 yo and vaccines are not licensed for adults over 45 yo [10]. Our study has data last available for 2018. Suppose there is a direct negative correlation between cervical cancer incidence and HPV vaccination. In that case, there should be decreasing cervical cancer rates among women 21- 57 yo with greater decreases among women 21- 38 yo. Across the entire 25-64 yo age group for the duration of the study, there was a -1.7 PC and insignificant APC. Considering there were an average of about 3,580 incident cases of cervical cancer per year, this yields an overall decrement of about 60 cases in 12 years. This is a weakly correlative result, which is reiterated graphically in Fig. (**2**) from the trend line y=-1.8736x + 7353 and R^2^ = 0.0061. Moreover, represented in Fig. (**2**), there is a dramatic fall followed by a rebound in cervical cancer incidence rates between 2008 and 2015. This trend could be attributed to a significant population change within the SEER registry area. The results from this study also vary significantly when separated by age group. There is a large counterintuitive outlier in the 30-34 yo age group which had a +13.8 PC to +1.5 APC. When separated by race, the non-Hispanic white population is the largest contributor to cervical cancer incidence. This groups’ incidences are higher than all other races/ethnicities combined. Therefore, since the NH white PC is -12 and APC is -0.7, these results could be attributed to the HPV vaccine program. However, if that is the case, the HPV vaccination program has adversely affected cervical cancer incidence in the non-Hispanic American Indian/Alaska Native population with a PC of +8.3, the non-Hispanic Asian/Pacific Islander population with a PC of +6.8 and the Hispanic population with a PC of 13.

### 4.1 HPV Vaccine Efficacy Based on Selected Alternative Endpoints

HPV vaccine efficacy based on alternative endpoints is generally excellent. The most consistent and arguably most impactful are HPV genotype-specific endpoints related to any anogenital source. Based on nucleic acid testing, all three HPV vaccines have shown between 90-100% efficacy for the specific HPV oncotype. A meta-analysis by Harper and Demars [3], “HPV vaccines - A review of the first decade”, reported efficacy against a cervical HRHPV 16/18 infection to be 94% and 96% for Cervarix and Gardasil respectively. Gardasil 9 had similar efficacies against the additional 5 HRHPV types, with 95-100% against 31/33/45/52/58.^3^ Multiple clinical trials boast between 94% and 100% efficacy for the respective vaccine oncotype against cervical infection. Non-cervical endpoints have close to 100% efficacy across any vulvar and vaginal intraepithelial neoplasia grade or cancer diagnosis concerning specific HPV types [1]. These high rates of efficacy against type-specific HPV have impacted the prevalence of HPV types in the U.S. A decade after the vaccines’ introduction, the prevalence of HPV types 6, 11, 16, and 18 in the US has decreased by 86% among females aged 14 to 19 yo and 71% among females 20 to 24 yo [10].

Cytologic testing for cervical cancer (and precancerous state) does not test directly for the HPV virus, but screens for the cytologic abnormalities that occur as a result of all HPV infections. Therefore, vaccines have lower efficacy rates for preventing these secondary endpoints, which detect dysplastic and neoplastic states, regardless of HPV genotype. According to Harper and Demars [3], prevention of abnormal cytology graded atypical squamous cells of undetermined significance (ASCUS) and higher, is variable across the three vaccines: Gardasil = 17%, Gardasil9 = 44%, and Cervarix = 27%. It is only HRHPV infections that persist into high grade neoplasia that transforms into cancer. In preventing high grade intraepithelial lesions (HSIL) specifically, the vaccines understandably perform better, with Gardasil preventing 45% of cases, Cervarix preventing 59% of cases, and unavailable data for Gardasil9.

Cervical pathology also provides secondary endpoints to HPV infection. Pathological diagnosis is generally indicated in ASCUS through HSIL cytologic findings and is carried out through cervical colposcopy and conization. High grade lesion cytology results generally correlate with high grade surgical pathology results. It is no surprise then that HPV vaccine efficacy against cervical intraepithelial neoplasia grade 3 (CIN3) and cervical cancer is significant. One Finnish cancer registry-based study that includes 18,000 16 to19 yo women, found an HPV 16/18 vaccine efficacy of 66% in preventing CIN3+ [11]. Results from other sources vary considerably. Cervarix has 100% efficacy in preventing adenocarcinoma in situ (AIS) with any HPV type according to two different sources. Against CIN3+ and CIN2+ with any HPV type, Gardasil has 43% and 22% efficacy, respectively. In contrast, Cervarix was found to have 93% efficacy against CIN3+ with any genotype and 62% efficacy against CIN2+ with any genotype [3].

Antibody titers are a measure of the immune system response to the virus-like particles in the vaccine. They depend on the number and timing of vaccine doses. According to a three-dose regimen, robust antibody responses were found with all three vaccines. One study used two different immunoassays (9v-competitive Luminex immunoassay and pseudo-virion-based neutralization assays) to test quadrivalent and nine-valent seropositivity across a 60-month period. 77.5 to 100% of participants remained seropositive for each HPV genotype at the end of the study [1]. Other studies have noted significant differences between specific HPV genotypes. After a three-dose Gardasil vaccination, one study found almost 35% of women lost detectable HPV 18 antibody titers after 5 years, and 15% lost HPV 45 after 2 years [3].

### 4.2 Strengths and Limitations

With a total of over 46,000 incident cases of cervical cancer over a 12-year period and an approximate coverage of about 34.6% of the U.S. population, SEER provides an immense data set for evaluation [8]. This is the gold standard for a national epidemiological study. SEER Stat evaluations of cervical cancer incidence to investigate the effects of the HPV vaccines are not novel and prior studies have shown similar results [12, 13]. However, to the authors’ knowledge, this is the most up to date published report utilizing the SEER data available in April 2021.

The major limitation of this data set is time. Although there is a substantial amount of data, not enough time has passed since the introduction of the HPV vaccine for such data to evaluate vaccine efficacy sufficiently. HRHPV oncogenesis (infection, persistence, progression, and invasion) can take over a decade. This means we are only at the beginning of a measurable impact on cervical cancer incidence. This study did not explore all alternative non-cervical HPV vaccine endpoints, particularly the incidence of HPV-associated mucosal cancer and squamous cell carcinoma (SCC). SCC is an epithelial neoplasm with squamous differentiation exhibited by the formation of keratin with the presence of intercellular bridges [14]. In addition to being associated with 99% of cervical cancers, HPV is indicated in vulvar, vaginal, anal, and penile cancers, as well as oropharyngeal SCC. In 2020, the CDC published data that associates 35,900 U.S. cases of these cancer types with HPV on an annual basis. According to the same data, HPV is associated with 69% of vulvar cancers, 75% of vaginal cancers, 91% of anal cancers, 63% of penile cancers, and 70% of oropharyngeal cancers [15]. Exploration of SEER data regarding the incidence of these HPV-associated cancers is warranted.

Additionally, this study did not investigate any possible correlation between vaccination rates among age and/or racial groups and cervical cancer incidence data. Data from the 2013-2018 National Health Interview Survey (NHIS) cites an increase from 13.8% to 21.5% of adults 18 to 26 yo who received the recommended number of HPV vaccine doses. The same data reports that, in 2018, vaccination rates were highest among women and non-Hispanic white adults [16]. The design of this descriptive study is also a limitation. This study explores trends over time for the entire U.S. population and assumes that the implementation of the HPV vaccine would affect cervical cancer incidence. It does not compare vaccinated and unvaccinated populations using randomized controlled trials. It is, therefore an ecological study.

It is clear from the results of this study, that trends in cervical cancer incidence mined from the SEER database should not be solely attributed to the implementation of the HPV vaccine program. Moreover, these results do not compare well with the efficacious alternative-based endpoints. There are multiple factors that could be responsible for the variations in incidence rates across ages and racial/ethnic groups. The results could reflect changing trends in sexual activity, safe sex practices, health-seeking or sexual behavior, geographic population flux, HPV genotype populations, cervical cancer reporting, cytological screening programs, or a number of unforeseen factors. One concern brought to light by this research, which could also be attributed to these factors, is the dip in cervical cancer incidence between 2008 and 2015. More research is needed to characterize this anomaly.

Lastly, this analysis concentrates on cancer prevention, not vaccine safety or hesitancy. The rare but most commonly reported physical side effect of the HPV vaccine is pain, possibly due to the virus-like-particles inflammatory process, but overall HPV vaccine hesitancy is most commonly associated with a lack of quality and amount of HPV vaccine information as well as a mistrust of healthcare administration [17, 18]. For example, in a small sample size of Hispanic women, the fear of side effects and risk perception was a barrier to getting vaccinated [19]. Further investigation into these correlations is warranted, as patients seek more information as they make vaccination decisions [20].

## CONCLUSION

It will take years for the results of a large population-based cervical cancer study, such as this one, to adequately characterize the efficacy of the HPV vaccine. Therefore, this data must be periodically updated and analyzed, especially given the possible adverse effects of the vaccines [21, 22]. While, HPV vaccines are a powerful public health tool in eradicating cervical cancer, it is important to emphasize the results of alternative endpoints, such as type-specific HPV genotyping in proving the ability of vaccines to prevent HRHPV infection and, subsequently cervical cancer. Furthermore, updated vaccine efficacy based on population-based cervical cancer studies could help overcome HPV vaccine hesitancy.

## Figures and Tables

**Fig. (1):**
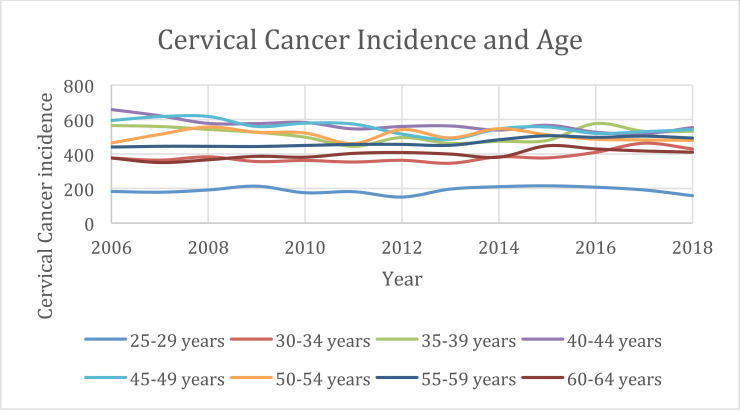
Annual cervical cancer incidence from 2006-2018 as a function of age; 25-64 years in 5 year increaments.

**Fig. (2):**
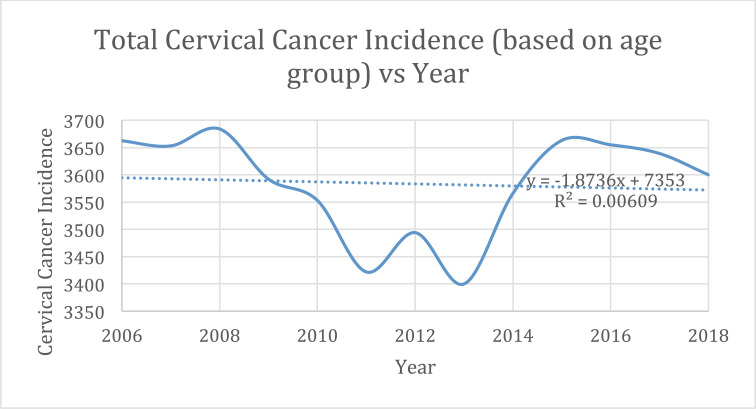
Annual cervical cancer incidence from 2006-2018 as a funtion of age with trend line. y = -1.8736x + 7353; R2 = 0.0061.

**Fig. (3):**
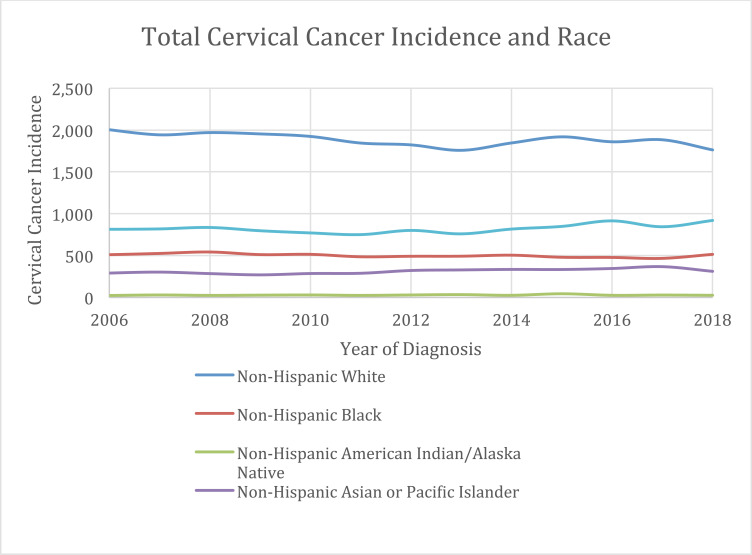
Annual cervical cancer incidence from 2006-2018 as a function of race (within 25-64 years old age range).

**Table 1A:** Frequency tables of cervical cancer incidence reported annually from 2006-2018 for 5-year age increments from 25-64 years old, total age 25-64 years old (within 25-64 years old age range).^9^

**Year of Diagnosis *vs* Age Increment**									
**Year**	**25-29 yo**	**30-34 yo**	**35-39 yo**	**40-44 yo**	**45-49 yo**	**50-54 yo**	**55-59 yo**	**60-64 yo**	**Total**
2006	184	378	565	659	595	464	440	377	3,662
2007	179	365	560	622	617	514	445	351	3,653
2008	192	384	543	578	618	557	445	367	3,684
2009	214	357	526	577	560	527	444	387	3,592
2010	176	363	498	583	579	522	450	382	3,553
2011	182	354	445	546	574	461	456	404	3,422
2012	151	364	496	560	516	542	456	409	3,494
2013	197	347	462	563	486	494	451	400	3,400
2014	211	384	474	539	546	547	483	382	3,566
2015	216	378	479	567	557	512	506	448	3,663
2016	208	410	577	527	520	486	497	430	3,655
2017	192	463	532	518	530	482	504	418	3,639
2018	159	430	532	554	542	479	493	411	3,600
2006-2018	2,461	4,977	6,689	7,393	7,240	6,587	6,070	5,166	46,583

**Abbreviation:** Yo = Years old.

**Table 1B:** Frequency tables of cervical cancer incidence reported annually from 2006-2018 for 5-year age increments from 25-64 years old, total age 25-64 years old, race (within 25-64 years old age range).^9^

**Year of Diagnosis *vs* Race in Selected 25 - 64-Year-Old Age Group**						
**Year**	**Non-Hispanic White**	**Non-Hispanic Black**	**Non-Hispanic American Indian/Alaska Native**	**Non-Hispanic Asian or Pacific Islander**	**Hispanic (All Races)**	**Total**
2006	2,003	510	24	292	814	3,643
2007	1,943	526	30	303	819	3621
2008	1,970	543	25	285	836	3659
2009	1,954	512	28	270	797	3561
2010	1,924	515	30	286	771	3526
2011	1,845	486	25	289	751	3396
2012	1,823	492	30	322	801	3468
2013	1,758	493	34	329	760	3374
2014	1,846	505	26	335	817	3529
2015	1,919	480	44	334	849	3626
2016	1,860	478	26	346	915	3625
2017	1,884	467	29	368	845	3593
2018	1,763	515	26	312	920	3536
2006-2018	24,492	6,522	377	4,071	10,695	46,157

**Table 2A:** Percent change of cervical cancer incidence reported annually from 2006-2018 for 5-year age increments from 25-64 years old, total age 25-64 years old, and race (within 25-64 years old age range).^9^

**Percent Change for Age and Race**									
Age range (years)	25-29	30-34	35-39	40-44	45-49	50-54	55-59	60-64	25-64
2006-2018 PC	-13.6	13.8	-5.8	-15.9	-8.9	3.2	12	9	-1.7
Race	NH White	NH Black	NH AI/AN	NH A/PI	Hispanic	-	-	-	-
2006-2018 PC	-12	1	8.3	6.8	13	-	-	-	-

**Table 2B:** Annual percent change of cervical cancer incidence reported annually from 2006-2018 for 5-year age increments from 25-64 years old, total age 25-64 years old, and race (within 25-64 years old age range).^9^

**Annual Percent Change for Age and Race**																				
**-**	**-**	**CI**		**-**	**-**	**CI**		**-**	**-**	**CI**		**-**	**-**	**CI**		**-**	**-**	**CI**		**-**
**-**	**APC**	**L**	**U**	**CT**	**APC**	**L**	**U**	**CT**	**APC**	**L**	**U**	**CT**	**APC**	**L**	**U**	**CT**	**APC**	**L**	**U**	**CT**
Age range (years)	25-29				30-34				35-39				40-44				45-49			
2006-2018 APC	0.3	-1.6	2.1	2,461	1.5*	0.4	2.6	4,977	-0.4	-1.7	1	6,689	-1.4*	-2	-0.8	7,393	-1.3*	-2.1	-0.4	7,240
Age range (years)	50-54				55-59				60-64				25-64				-			
2006-2018 APC	-0.4	-1.4	0.7	6,587	1.3*	0.9	1.7	6,070	1.4*	0.6	2.1	5,166	-0.1	-0.5	0.4	46,583	-	-	-	-
Race	NH White				NH Black				NH AI/AN				NH A/PI				Hispanic			
2006-2018 APC	-0.7*	-1.2	-0.2	24,492	-0.7*	-1.2	-0.1	6,522	1.3	-1.7	4.5	377	1.8*	0.8	2.8	4,071	0.9*	0	1.8	10,695

**Abbreviations:** PC = Percent change, APC = Annual Percent Change, CI = Confidence intervals, L = Lower, U = Upper, CT = Count. Confidence intervals are 95% for trends. Percent changes were calculated using 1 year for each end point; APCs were calculated using weighted least squares method. *: APC significant (*p* < 0.05) NH = non-Hispanic, AI = American Indian, AN = Alaska Native, A = Asian, PI = Pacific Islander.

## Data Availability

The data supporting the findings of the article is available within the article.

## LIST OF ABBREVIATIONS

APC: Annual Percent Change
ASCUS: Atypical Squamous Cells of Undetermined Significance
eRA: Electronic Research Administration
HPV: Human Papillomavirus
SEER: Surveillance, Epidemiology and End Results
